# Association of gene expression with biomass content and composition in sugarcane

**DOI:** 10.1371/journal.pone.0183417

**Published:** 2017-08-17

**Authors:** Nam V. Hoang, Agnelo Furtado, Angela J. O’Keeffe, Frederik C. Botha, Robert J. Henry

**Affiliations:** 1 Queensland Alliance for Agriculture and Food Innovation, The University of Queensland, St. Lucia, Queensland, Australia; 2 College of Agriculture and Forestry, Hue University, Hue, Vietnam; 3 Sugar Research Australia, Indooroopilly, Queensland, Australia; Universidade de Lisboa Instituto Superior de Agronomia, PORTUGAL

## Abstract

About 64% of the total aboveground biomass in sugarcane production is from the culm, of which ~90% is present in fiber and sugars. Understanding the transcriptome in the sugarcane culm, and the transcripts that are associated with the accumulation of the sugar and fiber components would facilitate the modification of biomass composition for enhanced biofuel and biomaterial production. The Sugarcane Iso-Seq Transcriptome (SUGIT) database was used as a reference for RNA-Seq analysis of variation in gene expression between young and mature tissues, and between 10 genotypes with varying fiber content. Global expression analysis suggests that each genotype displayed a unique expression pattern, possibly due to different chromosome combinations and maturation amongst these genotypes. Apart from direct sugar- and fiber-related transcripts, the differentially expressed (DE) transcripts in this study belonged to various supporting pathways that are not obviously involved in the accumulation of these major biomass components. The analysis revealed 1,649 DE transcripts between the young and mature tissues, while 555 DE transcripts were found between the low and high fiber genotypes. Of these, 151 and 23 transcripts respectively, were directly involved in sugar and fiber accumulation. Most of the transcripts identified were up-regulated in the young tissues (2 to 22-fold, FDR adjusted p-value <0.05), which could be explained by the more active metabolism in the young tissues compared to the mature tissues in the sugarcane culm. The results of analysis of the contrasting genotypes suggests that due to the large number of genes contributing to these traits, some of the critical DE transcripts could display less than 2-fold differences in expression and might not be easily identified. However, this transcript profiling analysis identified full-length candidate transcripts and pathways that were likely to determine the differences in sugar and fiber accumulation between tissue types and contrasting genotypes.

## Introduction

Sugarcane biomass could play a very important role in supporting second generation biofuel production. On average, about 64% of the total aboveground dry biomass in sugarcane production is from the sugarcane culm, while the rest (~36%) is from the trash (leaves) (as reviewed in [1]). In the sugarcane culm-derived biomass, the major components are sugars (mostly sucrose) and fiber (cellulose, hemicellulose and lignin), as reviewed in [2, 3]. In our recent assessment on a diverse sugarcane population [4], these two components, together with other insoluble matters (all known as total solids) make up about 22–39% of the fresh weight, while on a dry biomass basis, the sugar content ranges from 29 to 64% and fiber content from 29 to 61%. Sugarcane sugars (as a food source) have long been used for biofuel production, and in recent years, sugarcane fiber (also referred to in the broader term, lignocellulosic biomass) has been emerging as an alternative option for biofuel production.

Biomass accumulation in sugarcane culms has been shown to be a very highly regulated and tightly connected process in which photosynthetic carbon is partitioned into sugar production or fiber deposition [5–10]. This means that if more carbon is diverted to fiber accumulation, cell-wall synthesis and internode elongation; less would be available for sucrose accumulation and vice versa [11]. The sugarcane culm acts as a carbon sink with both sucrose and fiber (cellulose/hemicellulose) syntheses, requiring a nucleotide sugar, UDP-glucose as a precursor, for a review, see [10]. Cellulose and hemicellulose, once deposited, rarely re-enter the cell’s metabolic process [8], whereas, sucrose has a complex and dynamic accumulation pathway in which it is rapidly synthesized, re-partitioned (into cell-wall polysaccharides) and turned over between the vacuole, the cellular metabolic and apoplastic compartments, depending upon developmental stages [5, 7, 8, 12–14]. The cellulose, hemicellulose and lignin of the fiber fraction are synthesized in different metabolic pathways but are physically linked to each other to form the plant cell-walls [15], and are therefore accumulated in the same target organs [16]. These fiber components are primarily deposited over time during internode elongation (~150 days), and during internode expansion in diameter (for at least 350 days). In an immature internode, around 50% of the incoming carbon goes to the fiber fraction (the rest is mostly partitioned into supporting protein production), whereas, in a more mature internode, only around 8% is diverted to fiber, while most of the remainder is partitioned into storage sucrose (i.e. 66% of that in internode 9) [11].

As the accumulation of sugar and fiber is tightly connected, understanding the regulation (i.e. at the transcriptional level) of their accumulation requires analysis of both fractions. In recent years, efforts have been made to gain more insight into the sugarcane culm transcriptome, particularly, the transcripts that are associated with the sugar and fiber accumulation. These have helped to define the regulation of the carbon flow in the sugarcane plant, as a whole, starting from the leaf (source tissue) and continuing along the culm (sink tissue), as well as various complex metabolic and physiological networks involved in this process [6, 8, 12, 17]. These transcriptome studies have included, for instance, genes expressed in maturing internodes [7], sugar transporters in maturing internodes [18], transcript differential expression in maturing culms [19], transcripts involving in cell-wall metabolism and development [20], transcripts related to cellulose synthase (CesA) and sucrose transporter gene families [14] and the culm transcriptome analysis of contrasting genotypes for lignin content [21–23]. Despite the lack of a reference genome or a complete transcriptome, these studies have provided valuable information about the transcriptome in the sugarcane culms and the expression patterns/regulation of sugar- and fiber-related transcripts.

The aims of this study were: (**i**) to conduct transcript differential expression analysis between the young and mature internodal tissues of the sugarcane plant, as well as between the contrasting low and high fiber sugarcane genotypes; and (**ii**) to identify candidate transcripts associated with the carbon partitioning between sugar and fiber components in sugarcane. The analysis was done by using a newly constructed transcriptome reference database which contains transcripts defined as different isoforms, to determine the important genes/transcripts involved in the accumulation of the major biomass components in corresponding samples. The transcriptome database was sequenced by PacBio isoform sequencing (Iso-Seq), as described in Hoang et al. (2017) [24], can be accessed under the NCBI TSA accession number GFHJ00000000.1; and hereafter is referred to as the Sugarcane Iso-Seq Transcriptome (SUGIT) database. Identifying the differential expressed transcripts between immature and mature internodes could suggest potential transcripts that may be involved in sugar and fiber accumulation along the sugarcane culm [25], while those differing between the low and high fiber (or sugar) content would highlight the differences in expression patterns between these two groups. The study set out to increase understanding of the regulation of carbon flux into the major components in the biomass and the genetic basis of these traits at the transcriptional level and support genetic improvement of sugarcane for fiber and/or sugar production.

## Materials and methods

### Sample selection and preparation

Analysis was performed on 20 internodal samples, belonging to 10 sugarcane genotypes, which were classified into low and high fiber groups (Table 1). These samples were derived from a population previously described in [4]. The RNA extracted from these samples was also used in the construction of the SUGIT database (mentioned earlier), which was employed in the transcript expression analysis in this study. In brief, 5 genotypes were chosen for each of the low and high fiber groups, and for each of the 10 genotypes, 1 top (young) and 1 bottom (mature) internodal tissue samples were collected. The young tissues were defined as the fourth internodes from the top, while the mature tissues as the third internodes from the bottom. For each internodal sample, 4 representative culms from the same genotype were pooled to form 1 biological replicate. Internodes from the pooled culms were harvested, immediately cut into 0.5 cm-thick slices, followed by the removal of the rind and diagonal separation of the remaining pith into small 0.5 cm cubes, using a pair of secateurs. The whole excision process took about 1 min before snap-freezing in liquid nitrogen, and then samples were stored at -80°C until RNA extraction. To avoid changes in the transcriptome due to the different collection times, the excision was conducted between 10 am to 2 pm on the same day. Prior to RNA extraction, frozen samples were pulverized, while kept frozen, into a fine powder in cryo-jars using a Retsch TissueLyser (Retsch, Haan, Germany), as described in [26]. The frequency of 25/S was used and the time was 1 min 30 s. Samples and the cryo-jars were kept in liquid nitrogen, except for when being ground in the TissueLyser. About 1 g of pulverized sample powder was used for RNA extraction.

**Table 1:** Sugarcane genotypes used in this study, including 5 low and 5 high fiber genotypes.

Code	Genotypes	%Fiber^a^	Type	Female^b^	Male^b^
**6**	QS99-2014	31.2	Commercial	QA88-1178	Q205
**1**	QC02-402	31.4	Commercial	QN91-295	Q200
**12**	KQB08-32953	32.5	Introgression	QBYN04-26042	QC91-580
**9**	Q200	37.6	Commercial	QN63-1700	QN66-2008
**8**	Q241	39.6	Commercial	Q138	SP72-4728
**17**	QBYN04-26041	45.0	Introgression	ROC25	YN2002-356
**11**	KQB07-23863	45.7	Introgression	KQ228A	MQB89-12554
**10**	QN05-803	47.7	Commercial	QN86-1659	Q142
**16**	KQB07-24739	48.2	Introgression	QBYC04-10559	N29
**20**	KQB09-20432	49.8	Introgression	KQ228A	QBYN05-10390

^**a**^ %Fiber on the total dry mass (total solids).

^**b**^ Parental genotypes

### RNA extraction

RNA extraction was conducted using a two-step protocol as described in [26] employing a Trizol kit (Invitrogen), followed by a Qiagen RNeasy Plant minikit (#74134, Qiagen, Valencia, CA, United States). The RNA quality, integrity and quantity were determined by a NanoDrop8000 spectrophotometer (ThermoFisher Scientific, Wilmington, DE, USA), and on a 2100 Agilent Bioanalyser with a plant RNA NanoChip assay (Agilent Technologies, Santa Clara, CA, USA).

### RNA-Seq and read data processing

About 3 μg of each of the 20 internodal RNA samples was used for indexed-library preparation (average insert size of 200 bp), employing the TruSeq stranded with Ribo-Zero Plant Library Prep Kit for total RNA library (Illumina Inc.). Each sample was sequenced in two lanes using an Illumina HiSeq4000 instrument to obtain 150 bp paired-end read data, at the Translational Research Institute, The University of Queensland, Australia. This led to more than 80% of the paired-end reads found to be overlapping. The read data was assessed by FastQC [27] for quality and adapter sequences. Read adapters and quality trimming was done in CLC Genomics Workbench v9.0 (CLC-GWB, CLC Bio-Qiagen, Aarhus, Denmark) with a quality score limit <0.01 (Phred Q score ≥20, equivalent to the accuracy of the base calling of 99%), allowing a maximum two ambiguous nucleotides, and removing reads below 75 bp. Only paired-end reads from each lane were kept for each sample and then concatenated into one data file prior to analysis. Since the sequencing resulted in un-balanced read data between the top and bottom internodal samples of each genotype, to reduce the sample size bias in the downstream analysis, the larger samples were down-sized using the seqtk toolkit [28], to obtain approximately uniform sample sizes across each genotype. Further quality control was conducted on the samples in each group and for all samples in the experiment. This was done by first performing counts-per-million (CPM) and then log2 data transformation of the of raw count matrix for each sample, using the script *Perl_to_R* from the Trinity package. This was used to assess the samples before analysis, and to remove any outliers or potential confounders within the replicates, which could cause batch effects [29], based on transcript expression level, the transcript pairwise comparisons, Pearson correlations and principal component analysis.

### Transcript profiling and differential expression analysis

The pipeline was adapted from the Trinity v2.2.0 package [30, 31], designed for transcript profiling and differential expression analysis without reference genome sequences at the transcript isoform level, employing the RNA-Seq by Expectation Maximization (RSEM) software v1.2.31 [32]. To estimate the abundance of each transcript isoform, the RNA-Seq data of each of the samples was aligned against the SUGIT database using the Perl script *align_and_estimate_abundance*.*pl*. The Bowtie v2.2.7 program [33] was used with options "*—no-mixed—no-discordant—gbar 1000—end-to-end -k 200*". The sam alignment output file was converted to a bam file and sorted by samtools-1.3.1 [34]. The sorted bam file was subjected to the program RSEM for quantification of transcript abundance at the isoform level by fractional correcting of read alignment based on the probabilities of the transcript isoforms the reads originally came from, using its iterative process [32]. The transcript abundance estimation result was used to build the matrix of raw read counts and normalized expression values, by the perl script *abundance_estimates_to_matrix*.*pl*. The normalized expression measures included fragments per kilobase of feature sequence per million fragments mapped (FPKM) [35] and transcripts per million transcripts (TPM) [36]. The read count matrix for each sample combination was parsed using the script *run_DE_analysis*.*pl* for transcript differential expression analysis. To identify the differentially expressed (DE) transcripts, a negative binomial model was used to determine the relationship of the mean and the variance for the dispersion estimation in the DESeq2 package [37]. This was run in a pipeline using script *analyze_diff_expr*.*pl*. This DESeq2 package was suitable for general, data driven parameter estimation [38], allowing the selection of differentially expressed transcripts through a dynamic range of data and considering the unbalanced sequencing depth of the different samples. This differential expression analysis pipeline employed R program v3.2.0 [39], with the Bioconductor v3.4 [40] and the following packages: limma [41], ctc [42], and Biobase [43]. Finally, transcripts with a false discovery rate (FDR) adjusted p-value ≤ 0.05 and mean fold change ≥2 were marked as being significantly differentially expressed between the two groups compared. The DE transcripts were clustered by the package cluster 2.0.4 [44] and ape [45], then graphed using the function heatmap.2 in gplots [46]. Lists of up-regulated and down-regulated transcripts were generated for each of comparison.

### Functional annotation of identified differentially expressed transcripts

For general function comparison, the DE transcripts were annotated against the Gene Ontology (GO) database, using Blast2GO v4.0.2 [47] with default settings. This used a separate BLASTX homology search result (BLAST+ v2.3.0) with maximum blast hits of 100 against the NCBI non-redundant protein database and an e-value of 1e-10. The GO terms were assigned to each of the DE transcripts, and then the GO terms for each up-regulated and down-regulated transcript sets were extracted, enriched and compared by WEGO [48]. Only enriched GO terms with a p-value cutoff of 0.05 (considered being significant from the Pearson Chi-square test) were used in assessing the over-represented GO terms between the up- and down-regulated transcript sets. For further functional analysis, all DE transcripts were subjected to the program Mercator for automated sequence annotation [49, 50] and functional classifications (bins) were assigned to the DE transcripts. This functional bin annotations were based on: **(1)** the BLASTX homology searches with a cutoff of 80% against the *Arabidopsis* TAIR Release 10, PPAP SwissProt/UniProt Plant Proteins, TIGR5 rice proteins and Uniref90; **(2)** the reverse PSI-BLAST (RPS-blast) searches against the Clusters of orthologous eukaryotic genes database (KOG), conserved domain database (CDD); and **(3)** InterProScan search against the protein domain databases. The functional bins were visualized and analysed by the ImageAnnotator module, MapMan v3.5.1R2 [51]. This annotation pipeline using Mercator and MapMan (termed as MapMan annotation) assigned the transcripts into the most appropriate bins and reduced the multiple times the transcripts were represented in many bins, which differed from the GO term annotations [51]. *Arabidopsis* and rice were used as the main sources of information, since these two are still amongst the best annotated plant genomes.

### Transcripts specifically involved in accumulation of sugar and fiber

DE transcripts that were potentially involved in the accumulation of the major sugar and fiber components, including cell-wall metabolism, carbohydrate metabolism, photosynthesis, and phenylpropanoid pathway were investigated by using the MapMan annotation bins.

### Validation of DE genes using quantitative real-time PCR (qPCR)

To validate the RNA-Seq differential expression, a reverse transcription followed by qPCR was conducted on 4 μg total RNA from each of 8 selected samples including 4 young and 4 mature tissues from 2 low fiber (QC02-401, Q200) and 2 high fiber (QBYN04-26041, QN05-803) genotypes. Three reference genes including glyceraldehyde-3-phosphate dehydrogenase (GAPDH), ubiquitin (UBQ) and clathrin adaptor complex (CAC) were selected for internal normalization, based on published results in literature [52–55]. These genes were elucidated, from the RNA-Seq data, by their stable ubiquitous expression across all the samples. Multiple reference gene normalisation method [56, 57] was applied to the qPCR quantification cycle (Cq) values, and analysed with qbase+ software (VIB, Flanders, Belgium). A total of 8 putative DE genes were chosen for qPCR experiments. Alignment to the putative gene with least symmetry to the multitude of sequence isoforms was ascertained with Clone Manager 9 (Sci-Ed Software, Denver, US). Primer design was accomplished using Primer3 software in NCBI/Primer-BLAST [58]. List of genes and primer sequences are disclosed in S1 Table. Reverse transcription was performed according to the Tetro cDNA Synthesis Kit protocol (Bioline Reagents, London, UK). The cDNA was quantified with the NanoDrop 8000 spectrophotometer (Thermo Scientific, Waltham, US), for equal loading of 100ng cDNA per reaction of each sample run in triplicate. SensiFAST SYBR Lo-ROX Kit (Bioline Reagents, London, UK) was performed as per manufacturer’s protocol on the ViiA7 System (Applied Biosystems, Waltham, US). Accuracy and reliability of results were validated using triplicate biological and technical controls, melt curve analysis and the MIQE guidelines [59]. RNA-Seq TMM-normalized FPKM expression data were correlated against the comparative-Cq qPCR normalised gene expression data, using Microsoft Excel 2013.

### Data analysis

All Venn diagrams were generated by the online tool [60]. All Linux-based analyses were performed at the High Performance Computing clusters (Flashlite and Tinaroo), hosted by the Research Computing Center, The University of Queensland, Australia [61]. Analyses using CLC-GWB were run on a CLC Genomics Server, nodes and CLC-clients at QAAFI, the University of Queensland, Australia. All other analyses, unless otherwise stated, were performed using the Data Analysis ToolPak in Microsoft Excel 2013 and RStudio ver.0.9.8/R ver.3.1.2 [39].

## Results

### RNA-Seq summary

To investigate the differential expression of transcripts between the young and mature tissues of the sugarcane culm, and between the low and high fiber genotypes, an RNA-Seq experiment was conducted. Two groups of 5 low fiber and 5 high fiber genotypes were used, in which, for each genotype, 1 top internodal tissue sample and 1 bottom internodal tissue sample were collected by pooling respective tissues from 4 different sugarcane culms. The total number of trimmed reads obtained for each sample ranged from 6 to 53 million (Table 2). The total number of RNA-Seq reads for all the samples from the low fiber group was 293 million, while that of the high fiber group was 148 million. This made the total read data used in this analysis 441 million reads. The percentage of reads mapped to the SUGIT database ranged from 70 to 82%. In most cases (except for genotype 6), the bottom internodal samples had a higher percentage of reads mapped to the references compared to the top internodal samples (mean of 79.5% and 74.2%, respectively). Amongst 107,598 transcriptome reference sequences, the proportion of the transcripts that had reads mapped to (FPKM>0) ranged from 57% to 76%. This result indicates the proportion of total active transcripts originating from the culm, as the SUGIT reference database was derived from leaf, internode and root tissues.

**Table 2:** Summary statistics of samples.

Low fiber genotypes			High fiber genotypes		
Samples	Trimmed reads	%Reads mapped	Samples	Trimmed reads	%Reads mapped
**T1**	18,547,122	70.76	**T10**	14,370,404	72.10
**B1**	20,000,000	78.06	**B10**	16,000,000	81.09
**T6**	12,471,756	74.20	**T11**	8,153,320	72.94
**B6**	13,000,000	71.51	**B11**	9,000,000	80.71
**T8**	27,639,164	76.69	**T16**	27,263,742	75.47
**B8**	29,000,000	80.78	**B16**	29,000,000	80.31
**T9**	34,796,280	73.64	**T17**	14,979,572	76.92
**B9**	34,920,646	80.44	**B17**	16,000,000	80.47
**T12**	49,728,150	76.07	**T20**	6,275,176	73.44
**B12**	53,000,000	81.64	**B20**	7,000,000	80.15
**Total**	293,103,118		**Total**	148,042,214	

T denotes the top internodal samples while B denotes the bottom internodal samples. The number represents the genotype code as listed in Table 1.

### Global expression analysis of sugarcane RNA-Seq data

**Fig 1:**
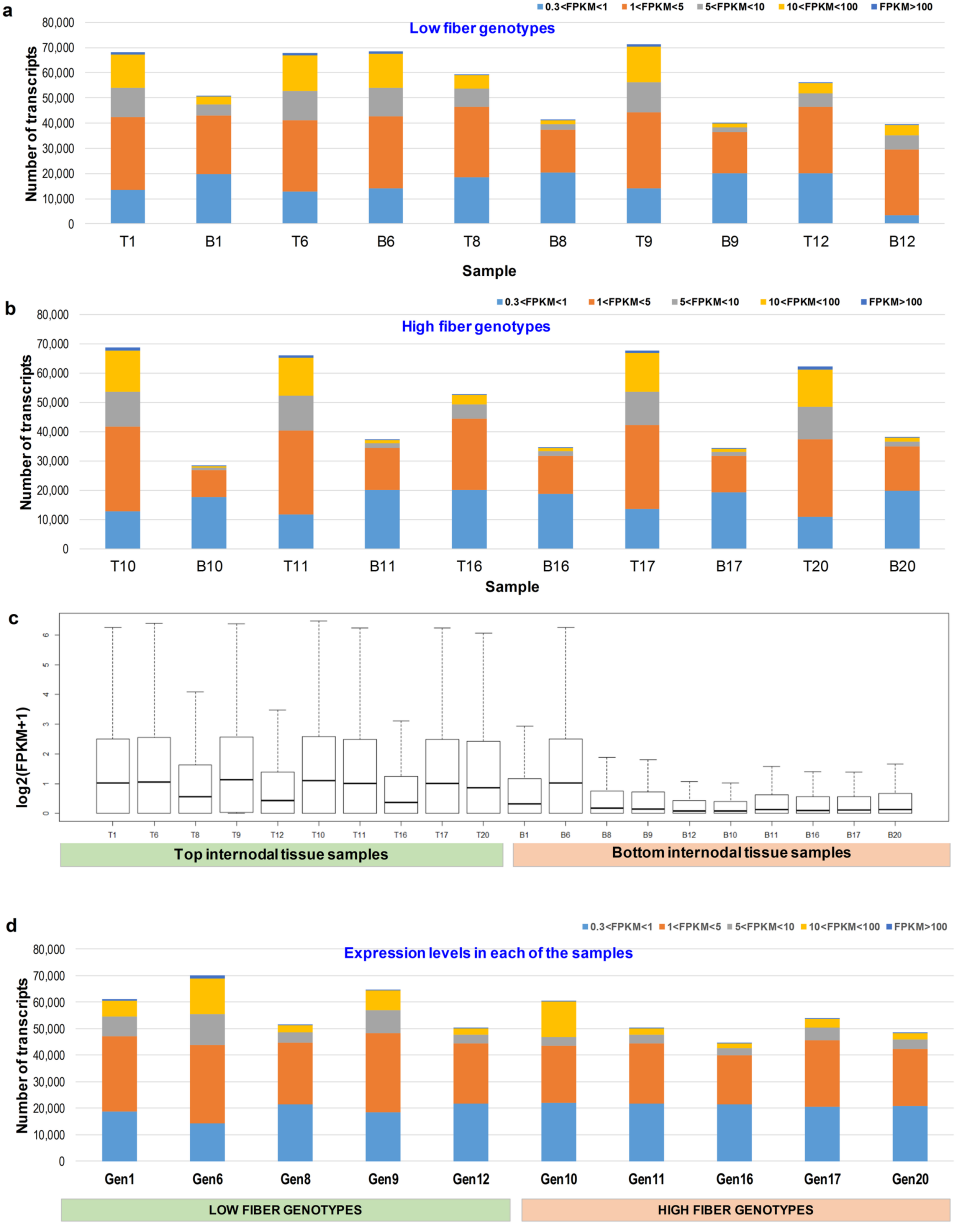
Global expression of samples used in this study. (**a**) Transcript expression of top and bottom internodal samples of low fiber genotypes. (**b**) Transcript expression of top and bottom internodal samples of high fiber genotypes. The x-axis indicates the samples while the y-axis represents the number of transcripts expressed. (**c**) Boxplot of log_2_(FPKM+1) distribution amongst the top and bottom internodal samples form low and high fiber genotypes. The x-axis indicates the samples while the y-axis represents the log_2_(FPKM+1). The transcripts with log_2_(FPKM+1)>6.5 were not shown here. T denotes the top internode while B denotes the bottom internode. (**d**) Global expression of 10 pooled genotypes. Gen denotes genotype.

In this analysis, all transcript isoforms were considered, but only transcripts with FPKM greater than 0.3 were counted as significantly expressed as suggested in [62] for RNA-Seq data. Overall, amongst the 20 samples studied, the number of transcript isoforms having FPKM ≥0.3 ranged from 29,339 to 71,156. The total profile of all transcripts expressed at FPKM of different ranges of 0.3–1, 1–5, 5–10, 10–100 and above 100, is presented in Fig 1a and 1b. As expected, there were more transcripts expressed in the top internodal samples (representing the young tissues) compared to that in the bottom internodal samples (representing the mature tissues). There was one exception in the case of genotypes 6 where a similar expression level in the top and bottom internodal samples was observed. This may indicate that the transcriptome expression was still similarly active along the culm in this genotype. In 8/10 genotypes, it was observed that the top internodal tissue samples had a lower proportion of transcript with 0.3<FPKM<1 (representing less active transcripts) and higher proportion of transcripts with FPKM>1 (representing more active transcripts) compared to the bottom internodal tissue samples. It can be seen from the distribution of log_2_(FPKM+1) in Fig 1c that even for the same tissue type, samples exhibited different expression patterns. Fig 1d shows the global expression of pooled data where the top and bottom internodal samples of each genotype were pooled into a single sample (representing a genotype) prior to mapping to the reference transcriptome. There was not a clear pattern in the total transcript expression level between two groups of low and high fiber genotypes. The total transcripts expressed at FPKM ≥0.3 ranged from 44,698 to 70,064.

**Fig 2:**
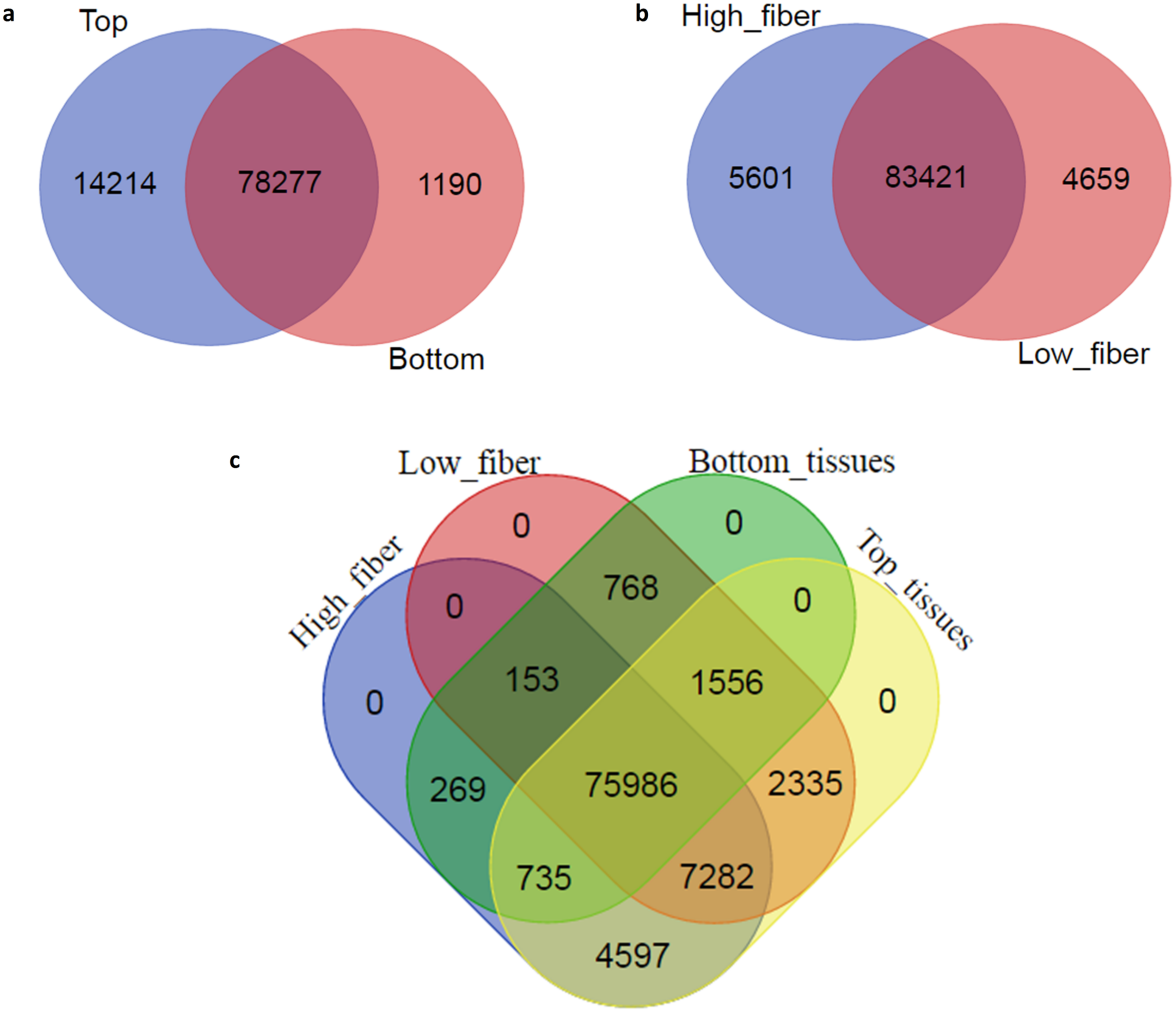
Comparison between the number of expressed transcripts (FPKM>0.3). (**a**) Between all top and bottom internodal samples, (**b**) All low and high fiber genotypes and (**c**) Between the four sample groupings.

Fig 2a shows that, of the total 93,681 unique transcripts found expressed in all top and bottom internodal samples, 78,277 were common between the two, while 14,214 and 1,190 were uniquely expressed in the top and bottom tissues, respectively. When the total unique expressed transcripts were separated according to low and high fiber genotypes (Fig 2b), the majority of transcripts (83,421) were found to be common between the two groups, only 4,659 and 5,601 transcripts were unique to low fiber and high fiber groups, respectively. In general, when all the samples were considered, there was a similar number of transcripts expressed in the two groups of low and high fiber genotypes in this study. Fig 2c reveals that 75,986 expressed transcript isoforms were common between the 4 fiber content-based and tissue-based comparisons. Taken together these results and data presented in Fig 1, it is suggested that each individual sample had a fraction of unique transcripts that was not detected in the others. Since these samples were derived from the same tissue type, at the global expression level, this could mean that, if one transcript was expressed in sample 1 but not detected in sample 2, it could be that it was not expressed at all in sample 2, or it could mean that it was down-regulated in sample 2 to a low expression level that was not detected (this could also be affected by a low sequencing depth).

### Transcript differential expression analysis

**Fig 3:**
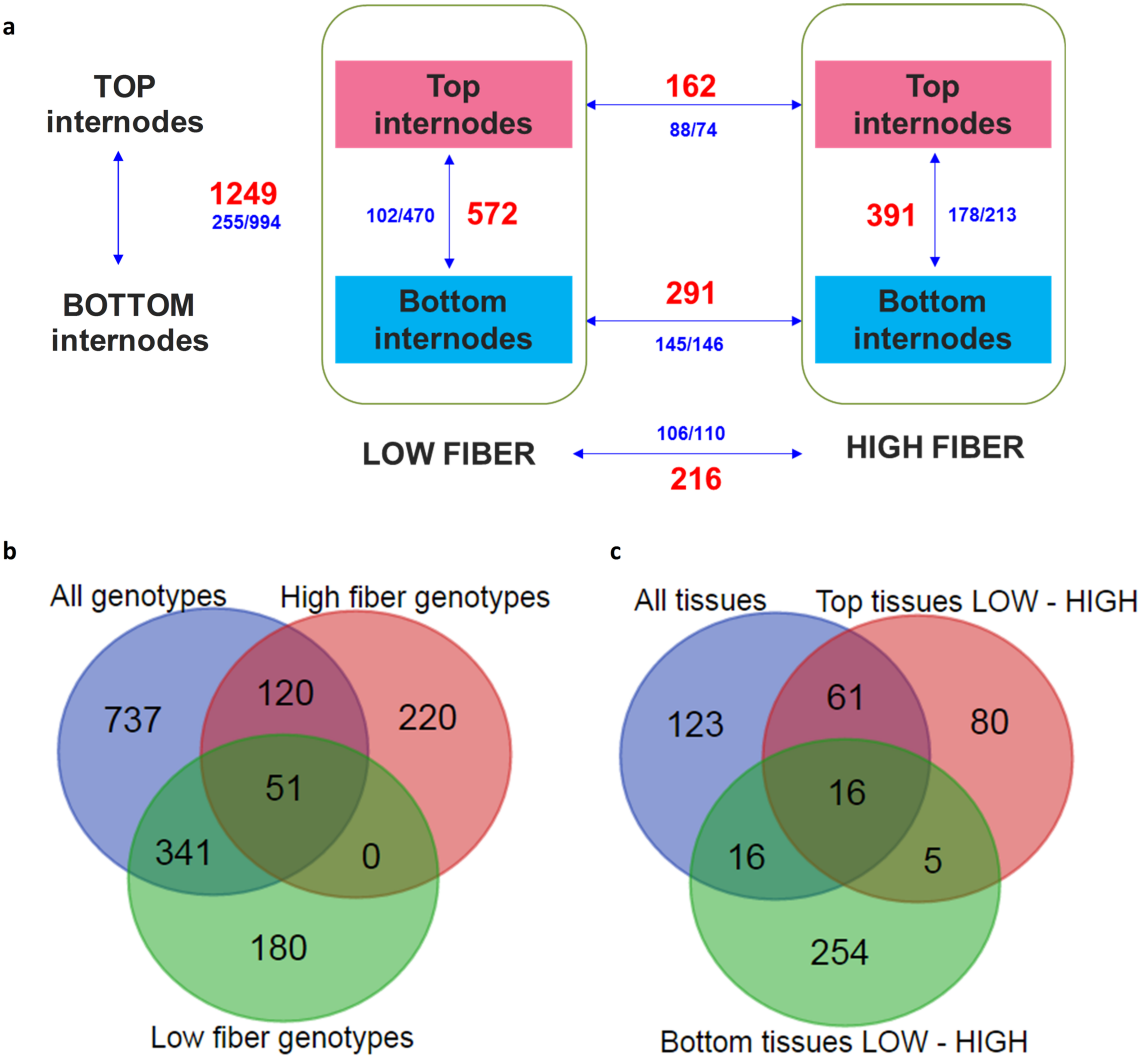
The summary of differentially expressed transcripts. (**a**) Differentially expressed transcripts. The red numbers denote the total DE transcripts, while the corresponding blue numbers denote the up-regulated/down-regulated transcripts. (**b**) Comparison among DE transcripts from all genotypes top vs. bottom tissues, low fiber genotypes top vs. bottom tissues, and high fiber genotypes top vs. bottom tissues. (**c**) Comparison of DE transcripts from pooled all tissues low vs. high fiber genotypes, bottom tissues low vs. high fiber genotypes, and top tissues low vs. high fiber genotypes.

The differentially expressed (DE) transcripts identified by the package DEseq2 with a fold change ≥2 and FDR adjusted p-value ≤0.05 were summarized in Fig 3a. In total, 1,249 DE transcripts were found between all 10 top internodal samples and all 10 bottom internodal samples (referred to as all-genotypes T-B); 572 DE transcripts between the 5 top internodal samples and the 5 bottom internodal samples of the low fiber genotype group (low-fiber T-B); and 391 DE transcripts between the 5 top internodal samples and the 5 bottom internodal samples of the high fiber genotype group (high-fiber T-B), respectively. When compared among these 3 sets of identified transcripts, 51 were found to be common among the 3 comparisons, 341 common only between all-genotypes T-B and the low-fiber T-B, and 120 common only between all-genotypes T-B and the high-fiber T-B (Fig 3b). Amongst the low and high fiber groups, a total of 216, 291 and 162 transcripts were identified as DE transcripts between the pooled tissues of low and high fiber genotypes (referred to as all-tissues L-H), between the bottom internodal samples of the low and high fiber genotypes (bottom-tissues L-H), and between the top internodal samples of the low and high fiber genotypes (top-tissues L-H), respectively (Fig 3c). Of all identified DE transcripts among the 3 comparisons of low and high fiber genotypes, 16 were found to be common among the three, 16 were common between only all-tissues L-H and bottom-tissues L-H, while 61 were common between only all-tissues L-H and top-tissues L-H.

### Transcript differential expression between the young and mature tissues in the sugarcane culm

**Fig 4:**
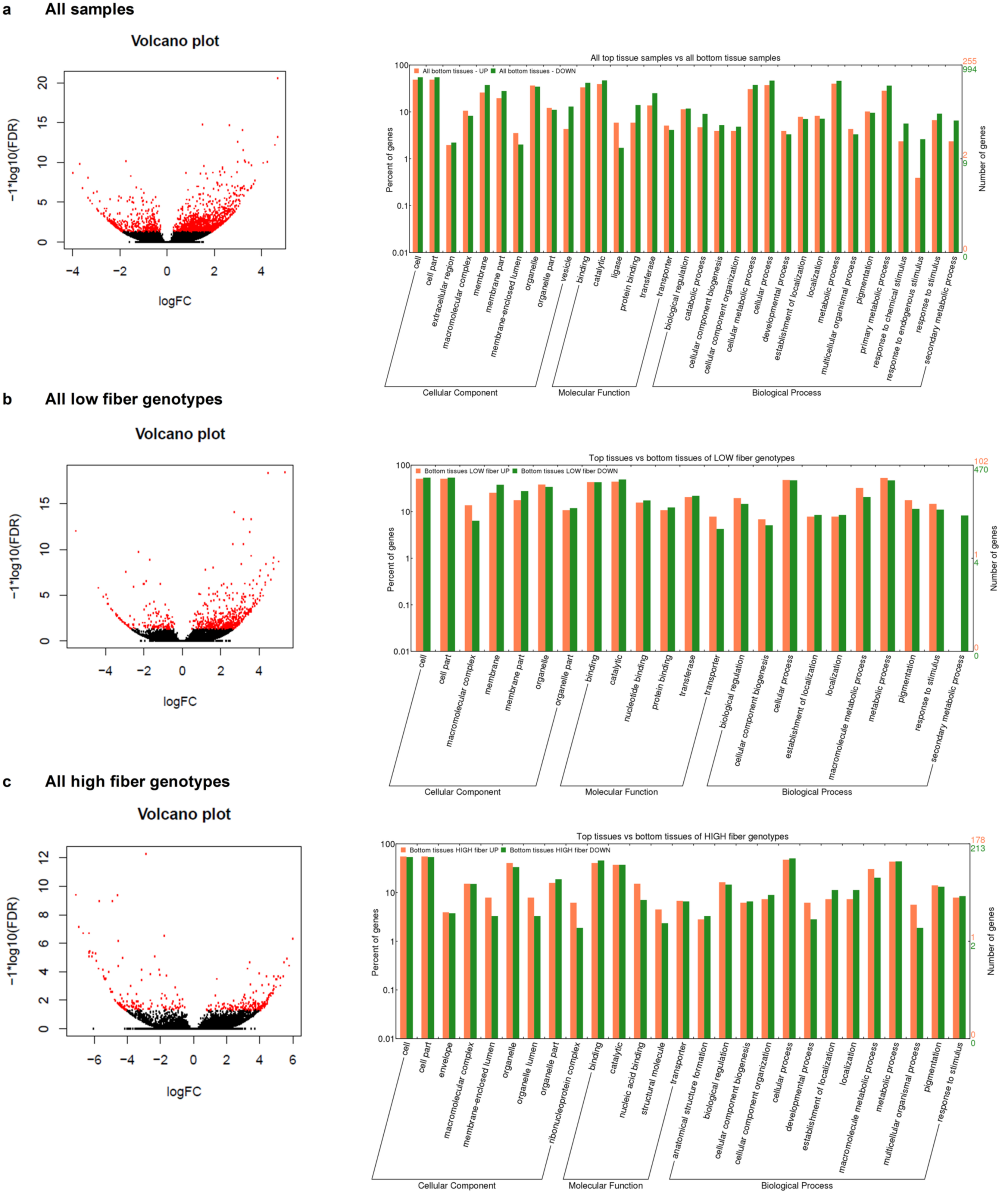
The volcano plots and GO term over-representation analysis between the top and bottom internodal tissues. (**a**) All genotypes, (**b**) Low fiber genotypes and (**c**) High fiber genotypes. In the left panel, the red dots denote the significant DE transcripts at FDR adjusted p-value <0.05, while the black dots denote the non-significant transcripts between the two cases. In the bar chart (right panel), the right y-axis indicates the number of transcripts for each GO term, while the left y-axis indicates the percentage of transcripts in each GO main category.

To provide an insight into the difference between the young and mature internodal tissues in the sugarcane culm, the identified DE transcripts derived from 3 tissue-based comparisons were investigated. The bottom internodal tissue samples were considered as the baseline (reference group) when compared to the top internodal tissue samples. Of the total 1,249 transcripts identified in the all-genotypes T-B comparison, 255 transcripts were up-regulated in the bottom tissues while 994 of those were down-regulated in the bottom tissues. Of 572 DE transcripts in the low-fiber T-B comparison, 102 and 470 transcripts were identified as up-regulated and down-regulated in the bottom internodal tissue samples, respectively. Of the 391 DE transcripts high-fiber T-B comparison, 178 and 213 transcripts were up-regulated and down regulated in the bottom internodal tissue samples, respectively. For all the 3 tissue-based comparisons, there were more down-regulated transcripts compared to up-regulated transcripts in the bottom tissues, which could be equivalent to the transcriptome in the top tissues being more active compared to that of the bottom tissues. Amongst all of the DE transcripts identified, there were transcripts with significant log2FC >4 (>16 fold) (Fig 4). In this analysis, since biomass accumulation is a highly regulated process that involves many quantitative trait loci (QTLs) [7, 14, 19, 22], and some of the transcripts were not well annotated, all GO terms that were associated with the up-regulation and down-regulation in the 3 comparisons were considered, enriched and highlighted the over representative functions in each comparison. This analysis revealed that the GO terms that were involved in the up-/ down-regulation between the top and bottom internodal tissues included those in various cellular components, molecular functions, and biological processes. The most abundant GO terms in the 3 comparisons were the cell and organelle part (in the cellular component category—CC), binding and catalytic (in molecular function category—MF), and cellular process and metabolic process (in biological process category—BP), shown in the right panel in Fig 4a–4c. When only significant GO terms that had p-value <0.05 were considered, in all-genotypes T-B comparison, the significant GO terms of the CC included those in vesicle GO:0031982 (down-regulated in the bottom tissues, hereafter referred to as “down”, otherwise, “up” for up-regulated), membrane GO:0016020 and membrane part GO:0044425 (down). Of the MF category, it was catalytic activity GO:0003824 (down), transferase activity GO:0016740 (down), ligase activity GO:0016874 (up), binding GO:0005488 (down), and protein binding GO:0005515 (down). Of the BP category, it was catabolic process GO:0009056 (down), secondary metabolic process GO:0019748 (down), cellular metabolic process GO:0044237 (down), primary metabolic process GO:0044238 (down), cellular process GO:0009987 (down), response to endogenous stimulus GO:0009719 (down), and response to chemical stimulus GO:0042221 (down). In the low-fiber T-B comparison, the significant GO terms were macromolecular complex GO:0032991 (down), membrane GO:0016020 (down), membrane part GO:0044425 (down) (in the CC); secondary metabolic process GO:0019748 (down) and macromolecule metabolic process GO:0043170 (down) (in the BP). In the high-fiber T-B comparison, the significant GO terms were membrane-enclosed lumen GO:0031974 (up), organelle lumen GO:0043233 (up), ribonucleoprotein complex GO:0030529 (up), organelle lumen GO:0043233 (up) (in the CC); nucleic acid binding GO:0003676 (up) (in the MF); macromolecule metabolic process GO:0043170 (up) and multicellular organismal process GO:0032501 (up) (in the BP).

The GO term analysis was consistent with the results in Mapman annotation (S2 Table and S1 Fig). Various classifications were found to be involved in the up- and down-regulated DE transcripts. Most function-assigned transcripts were down-regulated in the bottom internodal tissues or up-regulated in the top internodal tissues (blue color), apart from a proportion of DE transcripts were not assigned with a function (red color, bin 35, accounting for 27–32% of total DE transcripts). The most significant classifications included bins 29 (protein), 16 (secondary metabolism), 26 (miscellaneous enzyme families), 30 (signaling), 27 (RNA, including transcription factors), 34 (transport), 20 (stress), 10 (cell-wall related), 13 (amino acid metabolism), 11 (lipid metabolism), 31 (cell), and 17 (hormone metabolism). The important transcripts that were involved in sugar and fiber accumulation included those that were classified in bins of photosynthesis (bin 1), carbohydrates (bin 2 and 3), cell-wall metabolism (cellulose, hemicellulose and sugars—bin 10), and secondary metabolism (including lignin pathway—bin 16).

There were 151 transcripts (within the total 1,649 unique DE transcripts from 3 comparisons) that were directly involved in the accumulation of sugars and fiber, as summarized in Table 3, details in S3 Table. Amongst the identified DE transcripts, there were transcripts associated with carbohydrate metabolism (9 transcripts), photosynthesis (25), cell-wall proteins (8), cellulose synthesis (29), hemicellulose synthesis (6), cell-wall modification (3), cell-wall precursors (6), lignin biosynthesis (58) and dirigent proteins (7). Notably, for cellulose-related transcripts, 24 transcript isoforms were annotated as CesA and CesA-like proteins and 4 as COBRA-like protein precursor. Hemicellulose-related transcripts were those associated with endo-1,4-beta glucanase, plant glycogenin-like starch initiation protein 1 (PGSIP1), GT43 family glycosyltransferases, IRX14 and IRX9 genes. The transcripts for cell-wall precursors included those of UDP-glucose 6-dehydrogenase (EC: 1.1.1.22), UDP-glucose/GDP-mannose dehydrogenase and UDP-XYL synthase 6 (UXS6). Notably, there were a number of transcript isoforms involved in the monolignol metabolism, including 18 transcripts for phenylalanine ammonia lyases—PAL (EC: 4.3.1.24) and PAL (EC: 4.3.1.25), 7 transcripts for 4-coumarate CoA ligase (4CL, EC: 6.2.1.12), 12 transcripts for cinnamoyl CoA reductase (CCR, EC: 1.2.1.44), 3 transcripts for p-coumaroyl shikimate/quinate 3-hydroxylase (C3H, EC: 1.14.13.36), 3 transcripts for caffeic acid/5-hydroxyferulic acid O-methyltransferase—(COMT, EC: 2.1.1.68), 2 for cinnamyl alcohol dehydrogenase (CAD, EC: 1.1.1.195), 10 for caffeoyl CoA O-methyltransferase (CCoAOMT, EC:2.1.1.104) and 1 for ferulate-5-hydroxylase (F5H, EC 1.14.13.-).

**Table 3:** Differentially expressed transcripts involved in the accumulation of sugars and fiber between the top and bottom tissues in the sugarcane culm, based on MapMan annotation.

Function classification	Number of DE transcripts
Lignin pathway	58
Cellulose synthesis	29
Photosynthesis	25
Carbohydrate metabolism	9
Cell-wall proteins	8
Dirigent proteins	7
Cell-wall precursors	6
Hemicellulose synthesis	6
Cell-wall modification	3

**Fig 5:**
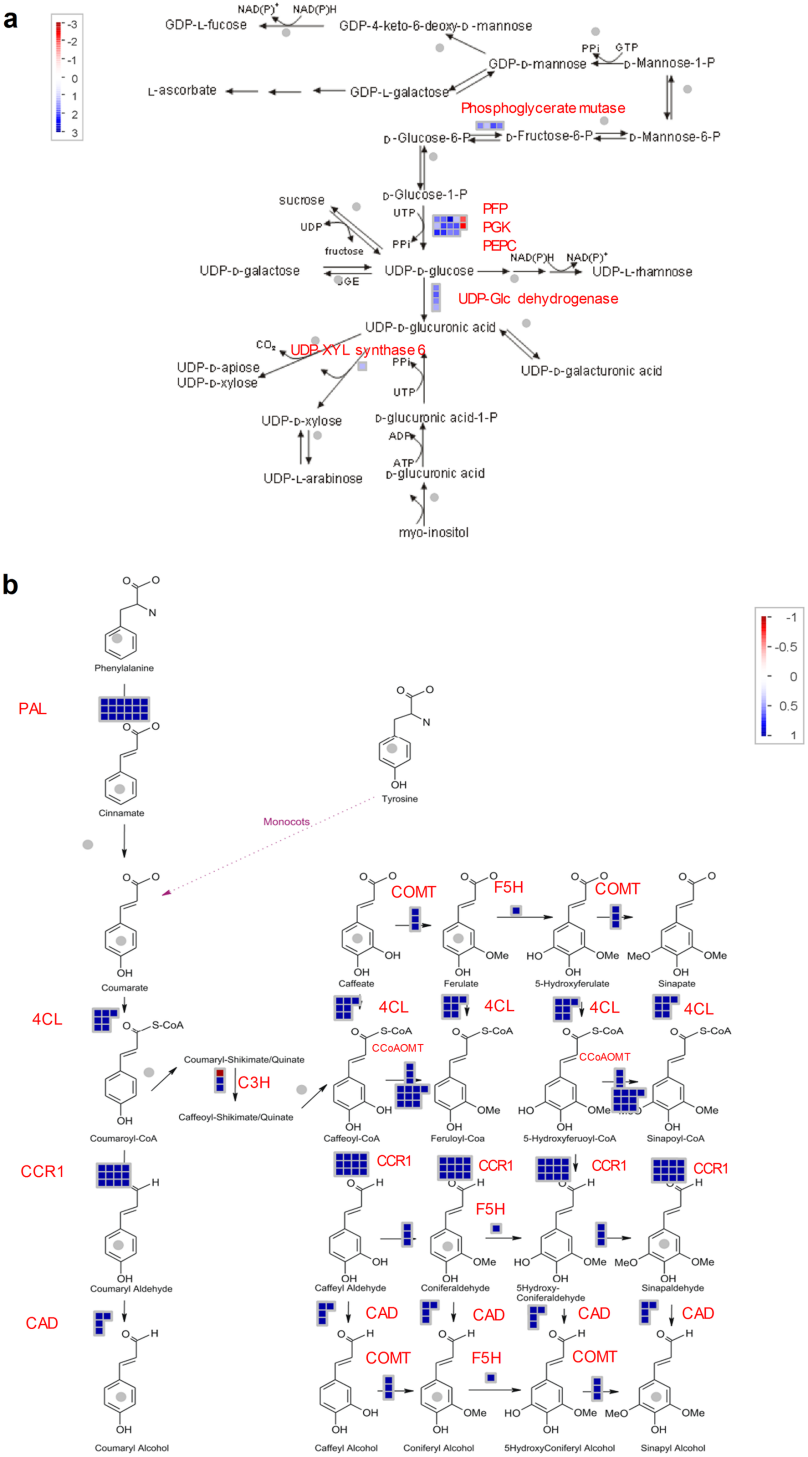
Differentially expressed transcripts between the top and bottom tissues in the sugarcane plant. (**a**) Cell-wall precursor metabolism. (**b**) Lignin pathway. Log_2_FC was used, red color denotes the up-regulated in the bottom internodal tissues while blue color denotes the down-regulated in the bottom internodal tissue. Colored boxes indicate the fold change. Each heatmap is representative of one of the identified differentially expressed transcripts.

Of the 151 identified transcripts related to sugar and fiber accumulation, all except 5 transcripts, were down-regulated in the bottom internodal tissue. There were 9 DE transcripts that were common among the 3 comparisons, including CesA, IRX9 gene, 4CL-1, two CCR isoforms, and four PAL isoforms. There were more DE transcripts that were common between all-genotypes T-B and only the low-fiber T-B comparisons (40 transcripts), than between all-genotypes T-B and only the high-fiber T-B comparisons (6 transcripts). Fig 5 shows the DE transcripts from the all-genotype T-B comparison, which involved 2 important pathways related to cell-wall precursor metabolism and the lignin pathway; while S2 Fig shows DE transcripts that were annotated as being involved in photosynthesis. The majority of the transcripts in these 3 pathways were down-regulated in the bottom internodal tissues / or up-regulated in the top internodal tissues samples.

### Transcript differential expression between low and high fiber sugarcane genotypes

**Fig 6:**
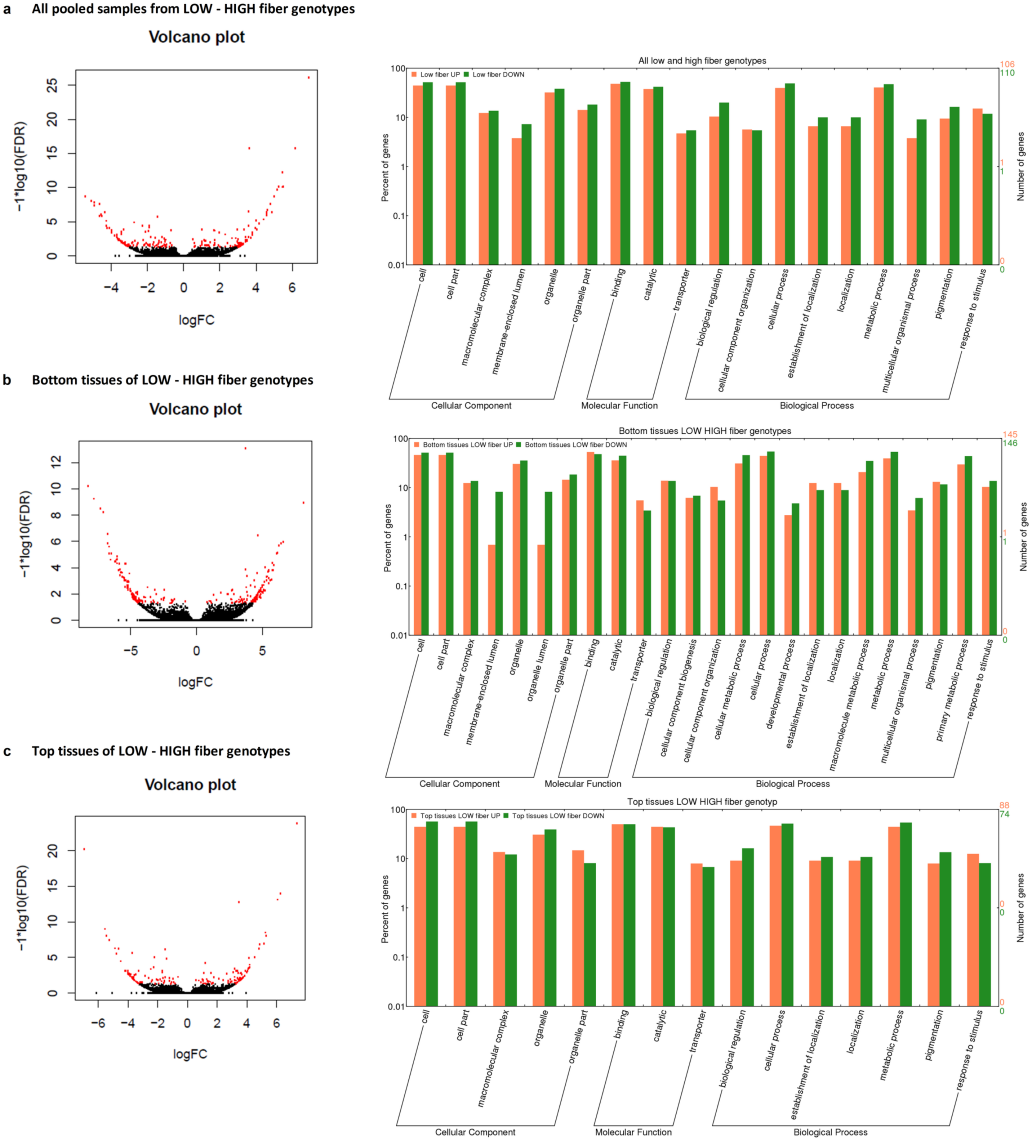
The volcano plots and GO term over-representation analysis between the low and high fiber genotypes. (**a**) All tissues from all genotypes, (**b**) Bottom tissues from low and high fiber genotypes, and (**c**) Top tissues from low and high fiber genotypes. The red dots denote the significant DE transcripts, while the black dots denote the non-significant transcripts between the two cases. In the bar chart (right panel), the right y-axis indicates the number of transcripts for each GO term, while the left y-axis indicates the percentage of transcripts in each GO main category.

In general, there were fewer DE transcripts identified in this fiber content-based comparison compared to that of the tissue-based comparison. Of 216 total identified DE transcripts from the pooled all-tissues L-H comparison, 106 transcripts were up-regulated and 110 were down-regulated in the bottom tissues of the low fiber group (or up-regulated in the high fiber group). Of the 291 DE transcripts from the bottom-tissues L-H comparison, 145 and 146 transcripts were up- and down regulated in the low fiber group. Of 162 DE transcripts from the top-tissues L-H comparison, 88 and 74 transcripts were up- and down-regulated, respectively, in the top internodal tissue samples of the low fiber group. Fig 6 summarizes the identified DE transcripts between the three comparisons, including those transcripts with significant log_2_FC >4. The GO analysis also indicated that identified DE transcripts were involved many cellular components, molecular functions and biological processes. This also suggests that the up/down-regulation also involved the same molecular functions and biological processes (Fig 6a–6c).

**Fig 7:**
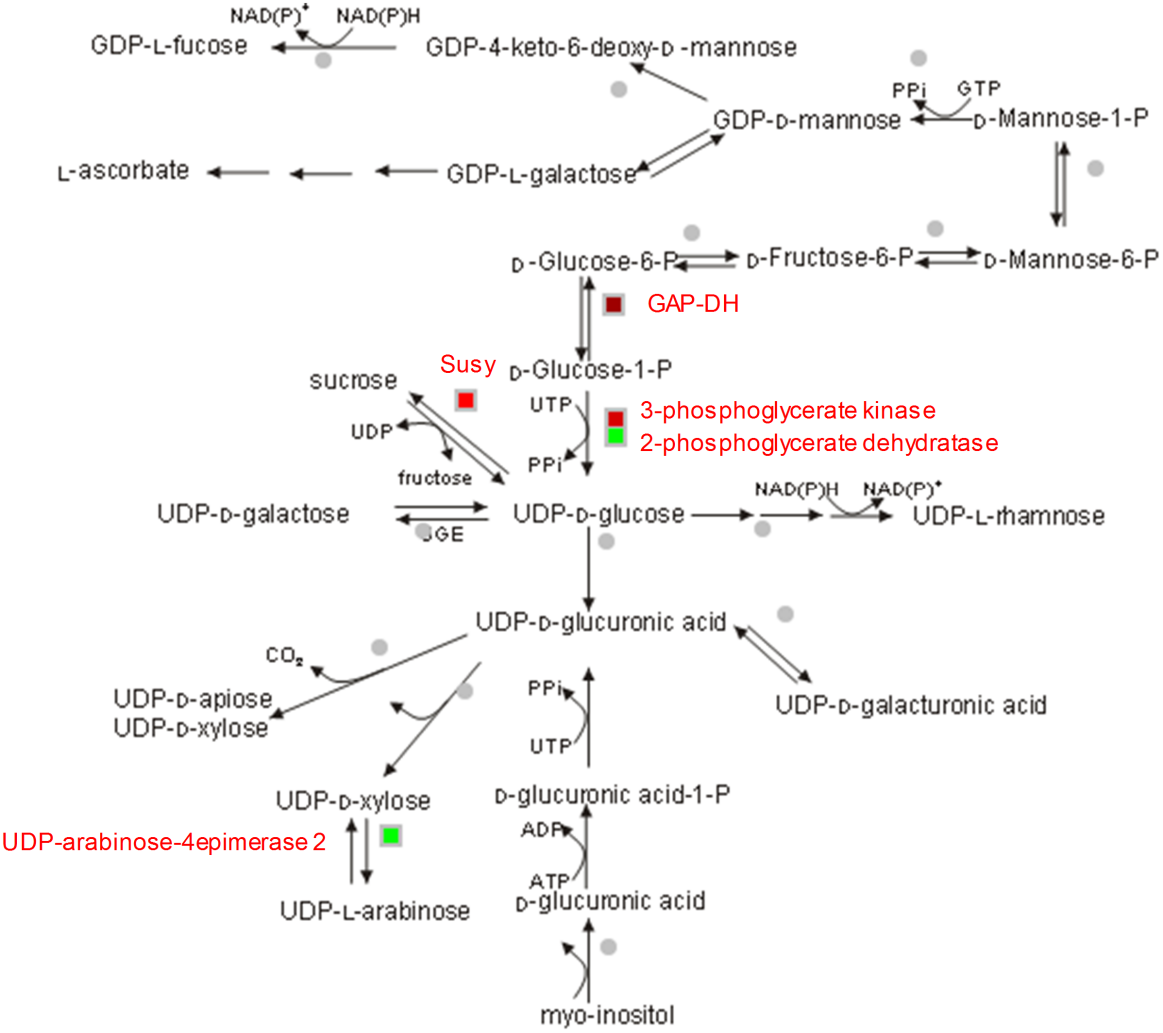
Differentially expressed transcripts involved in cell-wall precursor metabolism between the low and high fiber genotypes. Log_2_FC was used, green color denotes the up-regulated transcript in the low fiber group while red color denotes the down-regulated transcript in the low fiber group. Each heatmap is representative of one of the identified differentially expressed transcripts.

The significant GO term that had a p-value <0.05 in all-tissues L-H comparison was catabolic process GO:0009056, which belonged to the metabolic process in the BP category (up in low fiber genotypes). In the bottom-tissues L-H comparison, the significant GO terms were membrane-enclosed lumen GO:0031974 (down in the bottom tissues of the low fiber genotypes), organelle lumen GO:0043233 (down) (in the CC); metabolic process GO:0008152 (down), macromolecule metabolic process GO:0043170 (down), cellular metabolic process GO:0044237 (down), and primary metabolic process GO:0044238 (down) (in the BP). In the top-tissues L-H comparison, none were significant at p-value <0.05, due to a low number of DE transcripts. The Mapman annotation (S2 Table and S3 Fig) suggested that large proportions of the DE transcripts were attributed to bins 29 (protein), 27 (RNA), 20 (stress), 34 (transport), 16 (secondary metabolism), 31 (cell), 33 (development), 26 (miscellaneous enzyme families), 21 (reduction-oxidation regulation), 30 (signaling), 17 (hormone metabolism) and 13 (amino acid metabolism). A summary of the 17 transcripts that were involved in the sugar and fiber metabolism from MapMan annotation is presented in Table 4. These were derived from the total of 555 unique DE transcripts from the 3 fiber content-based comparisons. The up-regulated transcripts (in the low fiber genotypes) were callose synthase (GSL12) (log_2_FC = -3.031), alpha-amylase precursor (EC 3.2.1.1) (isozyme 1B) (log_2_FC = -3.223), photosynthesis helix protein (log_2_FC = -3.567), fructose-bisphosphate aldolase (log_2_FC = -4.497), UDP-arabinose 4-epimerase 2 (log_2_FC = -4.606), 2-phosphoglycerate dehydratase 1 (log_2_FC = -4.331), and CCoAOMT-5 (log_2_FC = -4.294). The down-regulated transcripts were glucose-1-phosphate adenylyltransferase large subunit 1 (log_2_FC = 1.050), granule-bound starch synthase 1b (log_2_FC = 2.525), sucrose synthase 1 (Susy, EC 2.4.1.13) (log_2_FC = 4.507), proteasome maturation factor UMP1 (log2FC = 3.575), serine hydroxymethyltransferase (logFC = 1.028), 3-phosphoglycerate kinase (PGK) (log_2_FC = 2.533), CesA6 (log_2_FC = 4.515), glyceraldehyde 3-phosphate dehydrogenase (GAP-DH) log_2_FC = 1.874, CAD (log_2_FC = 2.294), and COMT (log_2_FC = 6.619). In addition, 6 dirigent proteins were up-regulated in the high fiber genotypes compared to the low fiber genotypes, having been detected in both all-tissues L-H and bottom-tissues L-H comparisons. The transcripts associated with these proteins had a length ranging from 573 bp to 1,013 bp and with a log_2_FC ranging from 2.8 to 5.5 (8 to 30-fold). Fig 7 shows the identified DE transcripts involved in cell-wall metabolism.

**Table 4:** Differentially expressed transcripts involved in sugar and fiber accumulation between the low and high fiber genotypes.

Function classification	TSA accession	Length (bp)	Description	All tissues (1)		Bottom tissues (3)		Top tissues (2)	
				log_2_FC	padj	log_2_FC	padj	log_2_FC	padj
***Carbohydrate metabolism***									
CHO metabolism	GFHJ01020794.1	5097	Similar to callose synthase; glucan synthase-like 12 (GSL12)	-3.031	4.55E-02	-	-	-3.281	4.51E-02
CHO metabolism	GFHJ01023689.1	5963	Alpha-amylase precursor (EC 3.2.1.1) (Isozyme 1B)	-	-	-	-	-3.223	4.90E-02
CHO metabolism	GFHJ01037300.1	4596	Glucose-1-phosphate adenylyltransferase large subunit 1	1.05	3.79E-02	-	-	-	-
CHO metabolism	GFHJ01012375.1	1318	Granule-bound starch synthase 1b, chloroplast precursor (EC 2.4.1.242)	2.525	1.21E-02	2.676	2.45E-02	-	-
CHO metabolism	GFHJ01015836.1	2545	Sucrose synthase 1 (EC 2.4.1.13) (Sucrose-UDP glucosyltransferase 1)	-	-	4.507	3.77E-02	-	-
**Photosynthesis**									
Photosynthesis	GFHJ01093755.1	727	Encodes a one helix protein	-	-	-	-	-3.567	8.16E-03
Photosynthesis	GFHJ01031690.1	1385	Fructose-bisphosphate aldolase (ALDP) (EC 4.1.2.13)	-	-	-4.497	2.47E-02	-	-
Photosynthesis	GFHJ01079929.1	562	Proteasome maturation factor UMP1	3.575	4.31E-03	-	-	-	-
Photosynthesis	GFHJ01014544.1	1813	Serine hydroxymethyltransferase, mitochondrial precursor (EC 2.1.2.1)	1.028	5.19E-03	-	-	-	-
Photosynthesis	GFHJ01011598.1	1658	Phosphoglycerate kinase, cytosolic (EC 2.7.2.3)	-	-	2.533	2.29E-02	-	-
***Cell-wall metabolism***									
Hemicellulose synthesis	GFHJ01016359.1	1924	UDP-arabinose 4-epimerase 2 (EC 5.1.3.5)	-	-	-4.606	2.29E-02	-	-
Glycolysis	GFHJ01067367.1	835	2-phosphoglycerate dehydratase 1 (EC 4.2.1.11)	-	-	-4.331	3.77E-02	-	-
Cellulose synthesis	GFHJ01021631.1	1495	Encodes a cellulose synthase isomer, related to CESA6	4.515	1.57E-06	-	-	4.462	3.29E-05
Glycolysis	GFHJ01029222.1	1644	Chloroplast/plastid localized GAPDH isoforms	-	-	1.874	4.19E-02	-	-
***Lignin pathway***									
Lignin biosynthesis	GFHJ01052729.1	1079	Caffeoyl-CoA O-methyltransferase 5 CCoAOMT	-	-	-4.294	4.32E-02	-	-
Lignin biosynthesis	GFHJ01085183.1	589	Cinnamyl alcohol dehydrogenase (CAD)	2.294	3.60E-05	-	-	2.48	4.29E-04
Lignin biosynthesis	GFHJ01052435.1	1358	O-methyltransferase ZRP4 (EC 2.1.1.104) (COMT)	-	-	6.619	2.47E-06	-	-
***Dirigent proteins***									
	GFHJ01087645.1	573	Dirigent protein	3.665	3.24E-03	8.254	5.90E-11	-	-
	GFHJ01086725.1	741	Dirigent protein	5.454	1.96E-09	7.791	5.48E-10	-	-
	GFHJ01076697.1	754	Dirigent protein	3.431	9.29E-03	4.389	3.66E-02	-	-
	GFHJ01071693.1	872	Dirigent protein	2.885	1.31E-02	3.235	4.19E-02	-	-
	GFHJ01078847.1	931	Dirigent protein	3.223	2.27E-02	5.211	4.92E-03	-	-
	GFHJ01086396.1	1013	Dirigent protein	3.368	1.21E-02	5.412	2.97E-03	-	-

Negative log_2_FC values indicate that up-regulated in the low fiber group, while positive log_2_FC values indicate up-regulated in the high fiber group. padj: adjusted p-value.

^(1)^ Between all pooled samples from low fiber genotypes and high fiber genotypes.

^(2)^ Between top tissues of low and high fiber genotypes.

^(3)^ Between bottom tissues of low and high fiber genotypes. Grey colour indicates the transcripts that were only detected in the pooled sample comparison or only in one comparison.

### Differentially expressed gene validation by qPCR

To confirm the gene expression measured by RNA-Seq analysis, 8 putative genes differentially expressed between the young and mature tissues and between the low and high fiber genotypes were validated by qPCR. These include genes encoding COBRA-like 5 protein precursor, CAD, CCR, CESA, CESA-like C5, dirigent protein, a putative family 43 glycosyl transferase (IRX9 gene) and sucrose synthase 1 (selected from Table 4 and S3 Table). The reliability of the RNA-Seq data was confirmed by qPCR of 8 selected DE genes, in which each selected gene was shown as a single band at their respective size (data not shown). A significant correlation (*r* = 0.54, *p<0*.*001*, *n* = 64, *df* = 62) was found between the two data sets (S4 Table).

## Discussion

This study represents an effort to identify the transcripts involved in sugar and fiber accumulation by conducting differential expression analysis between the young and mature internodal tissues in the sugarcane plant, and between two groups of low and high fiber genotypes. The comparison between the young and mature internodes could potentially pinpoint the transcripts that are associated with carbon partitioning to the major biomass components in the sugarcane culm over time. The comparison between the low and high fiber genotypes could reveal the transcripts associated with sugar and fiber accumulation between the two groups. It is important to note that the two groups of low and high fiber genotypes used in this study were respectively equivalent to two groups of high and low sugar genotypes, since on a dry biomass basis, these two components were negatively correlated [4]. Overall, 4 out of 5 genotypes in the low fiber group were commercial sugarcane genotypes, while 4 out of 5 genotypes in the high fiber group were high fiber genotypes derived by introgression of genes from the wild *Saccharum spontaneum* relatives. The fiber content in these genotypes ranged from 31% to 50% of total dry mass (total solids which include sugars, fiber and others in the sugarcane culm biomass).

### Transcript differential expression between the young and mature tissues

According to the literature, sugar and fiber (cellulose, hemicellulose and lignin) in sugarcane are largely developmentally regulated, and consequently, it is expected that genes/transcripts involved in their biosynthesis are also developmentally regulated. In the sugarcane culm, the cell elongation and primary cell-wall deposition commences in internode 1, followed by the deposition of secondary cell-wall in internode 2, and then suberisation and sucrose accumulation [20]. Lignification has also been shown to start early in internode 1 [63], and continues to increase until the fifth or sixth internode. After that, the lignin content appears to be similar between tissues [20, 22]. At the early developmental stage, the internode acts as strong sink for sucrose, supporting the cell-wall synthesis and cell expansion, without an increase in sucrose concentration [14]. The accumulation of sucrose happens later, once the elongation ceases in maturing internodes and reaches a maximum in mature internodes [63]. After this, a major increase in cell-wall thickening (internode elongation) and lignification in the maturing tissues has been reported [6]. It is, therefore, the transcript expression associated with these changes in the culm development that is expected to be regulated, accordingly.

Early studies on *Arabidopsis thaliana* showed that CesA1, CesA2, CesA3, CesA5, CesA6 and CesA9 function in the biosynthesis of the primary cell-wall [64, 65], while CesA4, CesA 7 and CesA8 take part in the biosynthesis of the secondary cell-wall [66, 67]. Most CesA transcripts are down-regulated in the mature internodes compared to the young internodes, which reflects their roles in this type of tissues of the plant [68], however, CesA3 and CesA5 were found to be up-regulated, which is thought to be necessary for cell-wall maintenance [14, 20] or could be for radial growth in the mature tissue. The pattern of expression of the CesA-like transcripts, on the other hand, being highly abundant in the immature tissues, have been reported to not follow the pattern observed for the CesA in the culm [20]. A recent study [14] suggests that primary cell-wall synthesis in the sugarcane culm could occur throughout the sugarcane culm but is particularly important in the storage parenchyma of the maturing culm, where the internode is fully expanded and actively storing sucrose.

In this study, most of the 151 identified DE transcripts directly involved in the sugar and fiber metabolism, were up-regulated in the top internodal tissues compared to the bottom internodal tissues. These transcripts included those associated with the major carbohydrates, photosynthesis, several CesA and CesA-like transcripts, hemicellulose, cell-wall proteins, cell-wall precursors, major enzymes in the lignin pathway and dirigent proteins. The up-regulation in this tissue-based comparison represents the active growth and metabolism in the young internodal tissues where all the transcripts involved were highly expressed, compared to the less active mature internodal tissues of sugarcane. The top internodal samples were derived from the fourth internodes from the top, while the bottom internodal samples were from the third internode from the bottom of 12-month old sugarcane plants. Considering that each sample was pooled from four different plants and that there was plant-to-plant transcript expression variation, this could mean that the top internodal samples represented both the immature and maturing tissues, while the bottom internodal samples could represent mature tissues. Therefore, there would be highly expressed transcripts that regulate the cell-wall synthesis, lignification and sugar accumulation in the top internodal samples, whereas, these would be less active in the bottom internodal samples where most processes would be stopped or slowed down. That would also explain the up-regulation of the dirigent proteins and down-regulation of only 5 transcripts in the top internodal tissues compared to the bottom tissues. The dirigent proteins are hypothesized to play roles in scaffolding of lignin and biosynthesis of lignan [19, 69, 70] and dirigent domain-containing proteins are involved in the patterning of lignin-based Casparian strip in the root of *A*. *thaliana* [71]. However, some studies argued that lignin biosynthesis may not be handled by dirigent proteins, since lignin is not optically active and lignin biosynthesis is chemically controlled [72–74]. These proteins and a group of lignin-related enzymes (PAL, COMT and CCoAOMT) were found to be up-regulated in the maturing tissues when compared to the immature [19].

### Differential expression between the low and high fiber genotypes

This study identified 23 transcripts (including 6 dirigent proteins), out of 555 unique DE transcripts between low and high fiber genotypes, which were directly involved in sugar- and fiber-related pathways. The fewer DE transcripts in the fiber content-based analysis compared to the tissue-based analysis could reflect that there were many DE transcripts between these 2 groups whose fold change was at a relatively low level and which was not detected as differential expression. The cutoff for differential expression in this study was set at a minimum of two-fold, while the increase/decrease in fiber content between the two groups of low and high fiber genotypes could be from a combination of many up- or down-regulations involving transcript isoforms at a fold change less two-fold cutoff, as in Fig 6. In the previous tissue-based analysis, the difference between the samples was attributed to the developmental stages in which some of the pathways were significantly active/inactive between the stages, while in this fiber content-based comparison, it could be that all pathways were comparably active between the two groups being compared, except those were identified as DE. These identified DE transcripts could reflect the most important enzymes and pathways that played key roles in the processes making the difference in the fiber content. These included a number of transcripts that involved in carbohydrate metabolism, photosynthesis, cell-wall metabolism, monolignol metabolism and dirigent proteins, as presented in Table 4. The other possibility could be that the selected genotypes were not very contrasting in terms of fiber content, resulting in a narrow difference between the expression of the low and high fiber genotypes. These factors, together with the multiple genes/transcripts controlling the accumulation of sugars and fiber as discussed earlier, could explain the low number of DE detected in this analysis.

In relation to carbohydrate metabolism, callose synthase and alpha-amylase were up-regulated in the top tissues of the low fiber compared to that of the high fiber genotypes, while granule-bound starch synthase 1b and sucrose synthase 1 (Susy) were down-regulated in the bottom tissues of low fiber genotypes. Callose synthases are known to regulate the biosynthesis of callose, a cell-wall polysaccharide found in many higher plant species. Callose is a β-1,3-glucan, and has important roles in many developmental processes, including cell division and growth, tissue differentiation, cell plate formation, pollen development, plasmodesmata and response to stress [75–77]. Alpha-amylase on the other hand, plays roles in the starch degradation in the plant, breaking down the starch for other enzymes to act [78]. It is active when the stored carbohydrates are diverted back to the metabolism when it is required for plant development. Granule-bound starch synthase 1b is responsible for synthesis of starch (amylose) and the final structure of amylopectin [79], while Susy is the major enzyme of sucrose metabolism in sugarcane. The results indicate that, while all metabolic processes were probably happening in tissues of both groups of low and high fiber genotypes, there could be more active processes related to hemicellulose synthesis and starch degradation in the top tissues of low fiber, and more of starch synthesis and sucrose metabolism-related processes in the bottom tissues of high fiber genotypes.

With respect to photosynthesis, there was one up-regulated transcript identified in the top internode of low fiber which associated with one helix protein homologous to cyanobacterial high-light inducible protein. Between the bottom tissues of low and high fiber genotypes, ALDP fructose-bisphosphate aldolase was up-regulated while phosphoglycerate kinase (PGK) was down-regulated in the bottom tissues of low fiber genotypes. ALDP fructose-bisphosphate aldolase is one of the enzymes of the Calvin cycle and is predicted to have the potential to control photosynthetic carbon flux and biomass yields [80], while PGK catalyzes 1,3-biphosphogrycerate and ADP to form 3-phosphoglycerate and ATP [81]. Taking this together with 25 transcripts that were annotated as being associated with photosynthesis in tissue-based analysis (see S1 Fig), it was surprising to detect transcripts belonging to photosynthesis pathway in these samples, since they were derived from the sugarcane culms, a mostly non-photosynthetic tissue, and the rind of the culms was removed during sample excision. None of the data in the literature implies that significant photosynthesis occurs in this type of tissue. Photosynthesis requires a functional photosystem (PS) II and PSI system, a carbon fixation and the reductive pentose phosphate (RPP) pathway [82, 83]. It is noteworthy that the RPP pathway is present in all plastids, which are found in sugarcane culms, as it is crucial for many vital reactions. Some of the enzymes detected are part of the RPP pathway and this could be explained in terms of their roles in converting imported triose-P to starch, lipids and other cellular constituents in the sugarcane culm [9]. However, there were some up-regulated photosynthetic enzymes detected in the DE analysis which belonged to the PSI and PSII, which cannot be explained without further research. It could also be that the photosynthesis was located in the green rind of the sugarcane culms which were not completely removed from the samples.

In the cell-wall metabolism, there were two transcripts (UDP-arabinose 4-epimerase and 2-phosphoglycerate dehydratase) up-regulated in the bottom tissues of low fiber genotypes. UDP-xylose 4-epimerase is the enzyme that catalyzes a reversible reaction between UDP-arabinose and UDP-xylose [84], while 2-phosphoglycerate dehydratase catalyzes the conversion of 2-phosphoglycerate to phosphoenolpyruvate [85]. CesA6 was found down-regulated in the top tissues of low fiber genotypes, and glyceraldehyde-3-phosphate dehydrogenase (GAP-DH) was down-regulated in bottom tissues of low fiber genotypes. CesA6 is critical for cell elongation [86]; and GAP-DH, which can be induced or repressed in sugarcane under abiotic stress, i.e. salt stress tolerance [87], was previously reported to be highly abundant in maturing culms [18]. This result indicates that, apart from the similarly active processes between the two groups, processes related to internode elongation and cellulose synthesis were more active in the top tissues of the high fiber genotypes compared to the top tissues in the low fiber genotypes, while those related to hemicellulose synthesis were more active in the bottom tissues of the low fiber genotypes compared to that of the high fiber genotypes.

The down-regulation of the dirigent proteins and two enzymes in the lignin pathway (CAD and COMT), together with the up-regulation of the CCoAOMT-5 in the low fiber genotypes, reflect their functions in lignin biosynthesis may not the same at a given developmental stage. CAD and COMT are involved in the final steps in the monolignol biosynthesis, while, COMT and CCoAOMT also take part in the production of phenolic compounds [21]. Therefore, while they were present in the lignin pathway, the up- or down-regulation of these enzymes could also be interlinked to other pathways. In comparison of the low and high lignin sugarcane genotypes, PAL-3 and C4H-1 were up-regulated in the low lignin genotype while COMT-1 was up-regulated in high lignin genotype [21]. Only two transcripts related to lignin, C4H and CCoAOMT, were found up-regulated in a study on 11 high-lignin guineagrass genotypes [88]. As discussed in [21], after gathering the results of differential expression of several lignin genes, it was suggested that lignin deposition might be regulated at the transcriptional level, but also the post-transcriptional levels and by enzyme catalytic activities. RNA-Seq data might not reflect all events in the lignin pathway, and therefore the studies at post-transcriptional levels (i.e. proteome and metabolome) would be required to understand further lignin metabolism in the sugarcane plant.

## Notes on variation amongst diverse genotype collection and validation of RNA-Seq results

**Fig 8:**
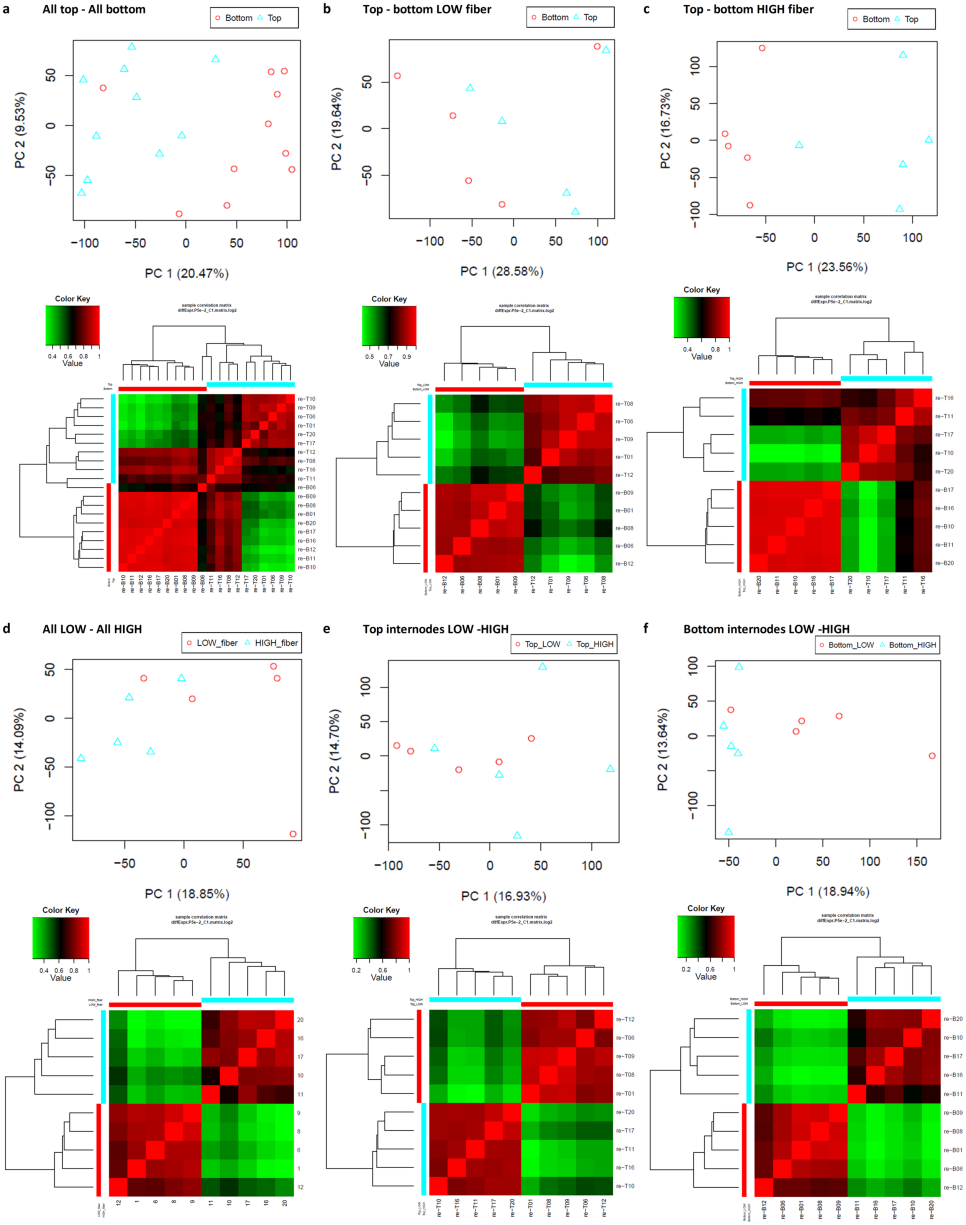
Principal component analysis (PCA) and correlation of expression patterns of the samples. This was based on all transcripts in the transcriptome dataset. (**a**) Between all top and all bottom internodal samples. (**b**) Between top and bottom internodal samples of low fiber genotypes. **c**, Between top and bottom internodal samples of high fiber genotypes. (**d**) All pooled low and high fiber genotypes. (**e**) Between the top internodal samples of low and high fiber genotypes. (**f**) Between the bottom internodes of low and high fiber genotypes. The value of correlation score was used.

Initially, a diverse collection of 40 samples (from 20 genotypes) were included in the design of this experiment, and it was hypothesized that even though there were dynamic expression patterns and noises within the sample groups of low and high fiber genotypes, the transcripts that play key roles in this difference would always be detected as up- or down-regulated consistently and distinct from the random noises. However, this hypothesis might only work with the traits of high heritability that are controlled by single genes or a few key genes, and with a detectable differential expression level, which can be distinguishable from sample noises. The DE transcript detection soft-wares or models, which are based on the FDR or p-values, normally favor the high fold change due to the un-certainty in reporting the DE transcripts of a low fold change, which could also be sample noises. Biomass traits such as those of sugar and fiber, as mentioned earlier, are controlled by a number of QTLs and the regulation is shown not to be straightforward. This could be reflected in the principal component analysis (PCA) of the transcript expression profiles of the samples. In this PCA, it is suggested that separating samples according to their tissues types (top–bottom tissues) or fiber content (low–high fiber) did not explain the major variation between the respective groups, and there were other factors/dimensions that contribute to the separation of the sample groups used in this study. It was shown in the scatter-plots (Fig 8), that the samples were not tightly clustered according to the tissue types nor fiber groups they belonged to. Overall, the separation according to their tissue types represented more the total variation in the data in comparison to that between the fiber groups, which agreed with the results where more DE transcripts were observed for the tissue-based comparisons than that of the fiber content-based comparisons. The first component (PC1) explained 20–29% of the total variation between the top and bottom internodal tissue groups, while the second component (PC2) accounted for 9–20% of the total variation. Between the low and high fiber groups, the PC1 explained 17–19% of total variation and the PC2 explained 13–15%. Notably, two groups of top and bottom tissue samples were separated into two clusters, except for samples from genotype 6 whose bottom internodal sample was situated in the same cluster as its top internodal sample (top panels Fig 8a and 8b). This was consistent with the global expression analysis which showed these two samples had similar expression patterns, which differed from the other genotypes whose bottom internodal samples had lower expression levels compared to the counterpart top internodal samples. There were less clear clusters between the two groups of low and high fiber genotypes. Overall, the two components explained about 30–48% total variation between the top and bottom internodal tissue samples, and ~32% of total variation between the low and high fiber genotypes. This agrees not only with the assumption that there were multiple factors responsible for the variation between the groups studied in this analysis, but also with the patterns in the dendrograms of Pearson’s pairwise divergence between the groups. The PCA and Pearson analysis could also suggest that there was variation amongst the samples in each group of tissue types or fiber content (bottom panels Fig 8a–8c and 8d–8f). This could be due to the fact that the genotypes were derived from different parental backgrounds, hence each of the genotypes had a unique expression pattern, possibly reflecting their chromosome number and the allelic contribution. The expression of each allele in the homologous group could vary in different environment, genotype or genotype-specific ripening time, which even when the samples were harvested at the same point of time since planting, might not have corresponded to developmental stage, transcriptome-wise. It would be also that the same fiber content in sugarcane results from the up- or down-regulation of different sets of transcripts, which could account for the same contribution towards the increasing/decreasing in fiber content. Using the correlation analysis, the samples whose expression patterns appeared to be very different from the rest in each group were removed, retaining the final collection of 10 genotypes for which the results in this study was based on.

Differentially expressed genes identified by microarrays or RNA-Seq experiments have often been confirmed by qPCR analysis [89, 90]. However, this has been shown to be difficult and un-reliable, especially in polyploid genomes, such as the allohexaploid wheat genome [91]. This is due to the fact that polyploids contain homo(eo)logous and paralogous gene copies, resulting in expression of different but related transcripts (i.e., different isoforms sharing the same exons/introns), which are not discriminated by the technique. It is also likely to be the case for the complex polyploid sugarcane genome, in which multiple homo(eo)logous gene copies (predicted up to 12 [92]), multiple promoter variants and transcript isoforms are likely to exist [14]. Our results showed a significant correlation (*r* = 0.54, *p* <0.001) between the RNA-Seq and qPCR results. This is the expected result as a very close correlation would not be expected due to the large number of different closely related genes and transcript isoforms in the highly polyploid sugarcane genome. This could result in amplification of multiple unknown copies of similar genes to the target in the PCR. Poor confirmation rates have been also reported in various studies on polyploid species with inter-platform discrepancies [91]. Designing a homo(eo)logous- and paralogous-specific validation array is complicated [14], RNA-Seq provides a more reliable way to analyse gene expression in a complex polyploid genome like sugarcane, due to the ability to distinguish the many closely related gene transcripts. The RNA-Seq analysis in this study involves a high degree of replication and sample pooling within each treatment providing strong statistical support for the differentially expressed genes identified.

## Conclusion

In this study, the newly constructed reference transcriptome, the SUGIT database, which was generated by the PacBio full-length isoform sequencing [24] was used for differential expression analysis. The results showed that 70 to 82% of the total RNA-Seq data to be aligned against this database, and 57–76% of the transcriptome had reads mapped to it, suggesting that the SUGIT database could be used as the reference database for transcript profiling and differential expression analysis. The proportion of RNA-Seq reads mapped is consistent with figures found in the literature, while the percentage of transcripts that had reads mapped indicated the proportion of transcripts that were expressed in the culm tissues out of the total transcriptome database derived from leaf, culm and root tissues.

The accumulation of sugar and fiber in sugarcane is a highly regulated process, and likely involves many genetic factors and QTLs. The differential expression analysis showed that there were many DE transcripts that were directly related to sugar and fiber metabolism, while others were not directly linked but could belong to supporting pathways. In this study, a total of 1,649 differentially expressed transcripts were identified, within which only 151 transcripts were directly related to sugar and fiber metabolism, when comparing between the young and mature internodal samples of the sugarcane culm. Most of these transcripts were up-regulated in the young internodes compared to the mature internodes, highlighting the growth phase of the two tissues being compared. When comparing the two groups of low and high fiber genotypes, there were a total of 555 differentially expressed transcripts identified, of which only 23 transcripts were related to the accumulation of sugars and fiber. This could indicate that the differential expression level between the two groups might be less than two-fold, and due to multi-factors contributing to fiber content and biomass accumulation.

## Supporting information

S1 Fig MapMan annotation of the DE transcripts between the top and bottom internodal tissues. (**a**) All genotypes, (**b**) Low fiber genotypes and (**c**) High fiber genotypes. Classification based on the MapMan annotation. PS denotes photosynthesis while CHO denotes carbohydrates. Red color indicates up-regulation while blue color indicates down-regulation in the bottom internodal tissues.
(XLSX)

S2 Fig Differentially expressed transcripts that were annotated as photosynthesis. Log2FC was used, red color denotes the up-regulated in the bottom internodal tissues while blue color denotes the down-regulated in the bottom internodal tissue. Each heatmap is representative of one of the identified differentially expressed transcripts.
(XLSX)

S3 Fig MapMan annotation of the DE transcripts between the low and high fiber genotypes. (**a**) All tissues from all genotypes, (**b**) Bottom tissues from low and high fiber genotypes, (**c**) Top tissues from low and high fiber genotypes. The classification was based on the MapMan annotation. PS denotes photosynthesis while CHO denotes carbohydrates. Blue color indicates up-regulation while red color indicates down-regulation in low fiber.
(XLSX)

S1 Table List of genes and primer sequences used in qPCR analysis. (XLSX)

S2 Table Percentage of transcripts annotated against MapMan functional bins of all comparisons in this study. (XLSX)

S3 Table List of identified transcripts that involved in the accumulation fiber and sugar between the top and bottom tissues of sugarcane plant. (XLSX)

S4 Table qPCR results and correlation analysis with the RNA-Seq data. (XLSX)
